# Co-occurrence of frameshift mutations in *SMAD6* and *TCF12* in a child with complex craniosynostosis

**DOI:** 10.1038/s41439-018-0014-x

**Published:** 2018-06-28

**Authors:** Andrew T. Timberlake, Robin Wu, Carol Nelson-Williams, Charuta G. Furey, Kristi I. Hildebrand, Scott W. Elton, Jeyhan S. Wood, John A. Persing, Richard P. Lifton

**Affiliations:** 1 Department of Genetics, Yale University School of Medicine, New Haven, CT USA; 2 Section of Plastic and Reconstructive Surgery, Yale University School of Medicine, New Haven, CT USA; 3 Division of Pediatric Neurosurgery, University of North Carolina School of Medicine, Chapel Hill, NC USA; 4 Division of Plastic and Reconstructive Surgery, University of North Carolina School of Medicine, Chapel Hill, NC USA; 5 Laboratory of Human Genetics and Genomics, The Rockefeller University, New York, NY USA

## Abstract

Non-syndromic craniosynostosis (CS) affects 1 in 2350 live births. Recent studies have shown that a significant fraction of cases are caused by de novo or rare transmitted mutations that promote premature osteoblast differentiation in cranial sutures. Rare heterozygous loss-of-function (LOF) mutations in *SMAD6* and *TCF12* are highly enriched in patients with non-syndromic sagittal and coronal CS, respectively. Interestingly, both mutations show striking incomplete penetrance, suggesting a role for modifying alleles; in the case of *SMAD6*, a common variant near *BMP2* drastically increases penetrance of sagittal CS. Here, we report a proband presenting with both sagittal and coronal craniosynostosis with the highly unusual recurrence of CS within two months of initial surgery, requiring a second operation to re-establish suture patency at six months of age. Exome sequencing revealed a rare transmitted frameshift mutation in *SMAD6* (p. 152 fs*27) inherited from an unaffected parent, absence of the common *BMP2* risk variant, and a de novo frameshift mutation in *TCF12* (p.E548fs*14). *SMAD6* and *TCF12* independently inhibit transcriptional targets of BMP signaling. The findings are consistent with epistasis of these mutations, increasing penetrance and severity of CS in this proband. They also add to the list of composite phenotypes resulting from two Mendelian mutations, and support the utility of exome sequencing in atypical CS cases.

The female proband was delivered at term after an uncomplicated pregnancy, and was referred to the pediatric neurosurgical service at birth due to her abnormal head shape. On physical exam, the patient had flattening of the left frontal bone with contralateral frontal bossing and associated harlequin deformity of the left orbit, consistent with left coronal synostosis. In addition, the calvarium posterior to the coronal sutures was elongated and narrow (scaphocephalic), consistent with sagittal synostosis (Fig. 1a, b). A CT scan confirmed sagittal and left coronal synostosis, and the child underwent endoscopic strip craniectomy at nine weeks of age.

**Fig. 1:**
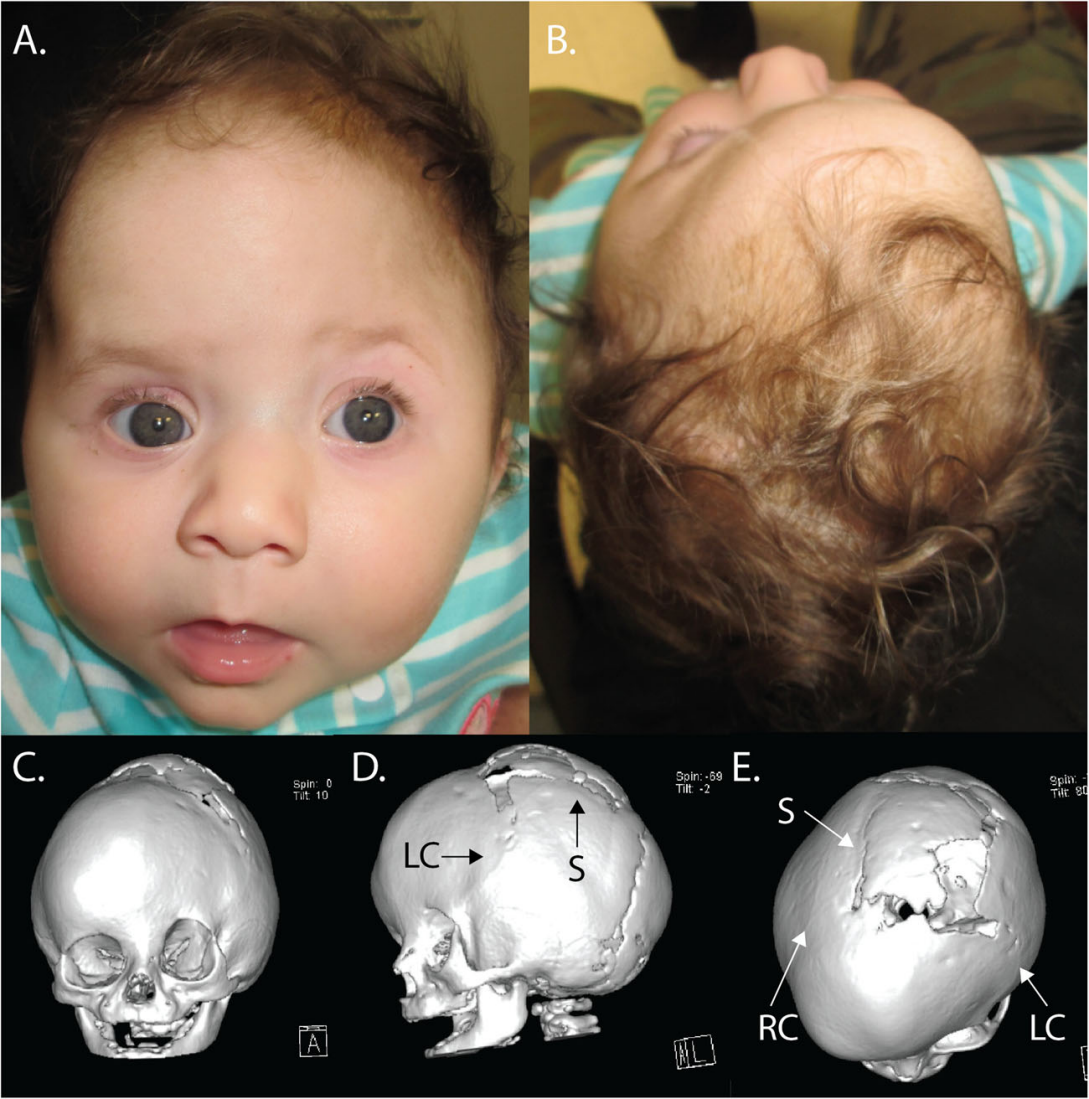
Patient photographs and 3D CT imaging from a second operation at 6 months of age. **a** Frontal view demonstrating left coronal synostosis with anterior plagiocephaly (suture fusion causing ipsilateral flattening and contralateral bossing of the forehead). Note the “twisted” appearance of the face as a result of unicoronal synostosis. **b** Anterior plagiocephaly as seen from above. Note the bossing of the right forehead and retrusion of the left forehead. Coronal (**c**), lateral (**d**), and axial (**e**) views of the 3D CT reconstruction performed three months after the initial strip craniectomy (age 5.5 months), demonstrating fusion of both the right coronal (RC) and left coronal (LC) sutures and sequelae of the previous strip craniectomy (S)

The child was subsequently followed biweekly in neurosurgery clinic and by an orthotist for helmet adjustments to shape skull growth. Two months after surgery, the orthotist noted that the child’s left forehead remained flattened and was not rounding as expected. At the child’s subsequent neurosurgical clinic visit, the parietal and occipital regions had rounded and expanded as expected; however, the left frontal region had stopped rounding. A repeat head CT was performed, which demonstrated rapid healing and patency of the sagittal suture and re-fusion of the left coronal suture along with complete fusion of the right coronal suture (Fig. 1c–e). Such rapid recurrence of craniosynostosis is extremely unusual. To correct the anterior deformity, the child underwent a cranial vault reconstruction with fronto-orbital advancement at six months of age. The child is developing normally to date, and it is unknown at present if she will need further surgical cranial reconstruction.

To explore potential genetic contributions to her condition, we performed whole exome sequencing of the case-parent trio using DNA prepared from buccal swab samples according to standard protocols. Exome capture was performed using the IDT xGen capture reagent, which was followed by 99 base paired-end sequencing on the Illumina HiSeq 2000 instrument. Sequence reads were aligned to the GRCh37/hg19 human reference genome using BWA-Mem. Local realignment and quality score recalibration were performed using the GATK pipeline, after which variants were called using the GATK Haplotype Caller. A Bayesian algorithm, TrioDeNovo, was used to call de novo mutations^1^. VQSR ‘PASS’ variants with an ExAC allele frequency ≤10^−3^ sequenced to a depth of eight or greater in the proband and 10 or greater in each parent with Phred-scaled genotype likelihood scores >30 and de novo quality scores (log_10_(Bayes factor)) >6 were considered. Independent aligned reads at variant positions were visualized in silico to remove false calls. All retained calls had de novo genotype quality scores of 100. Transmitted variants were called as per above, and all variants were annotated using ANNOVAR^2^ with allele frequencies assigned to each variant from the ExAC database^3^.

Analysis showed that the proband had rare heterozygous LOF mutations in both of the two predominant non-syndromic CS genes, *SMAD6* and *TCF12*^4,5^. The mutation in *SMAD6* was an early frameshift mutation (p. 152 fs*27), which was transmitted from an unaffected parent (Fig. 2, Table 1). The mutation in *TCF12* was also a frameshift mutation (p.K548fs*14), which was de novo. Both mutations were absent from the ExAC and GnomAD databases, which contain >240,000 alleles^3^, and both mutations were confirmed by Sanger sequencing (Fig. 2). No other compelling heterozygous rare LOF or damaging missense variants were identified, and no rare recessive genotypes were identified (Table 1, Supplementary Table 1).

**Fig. 2:**
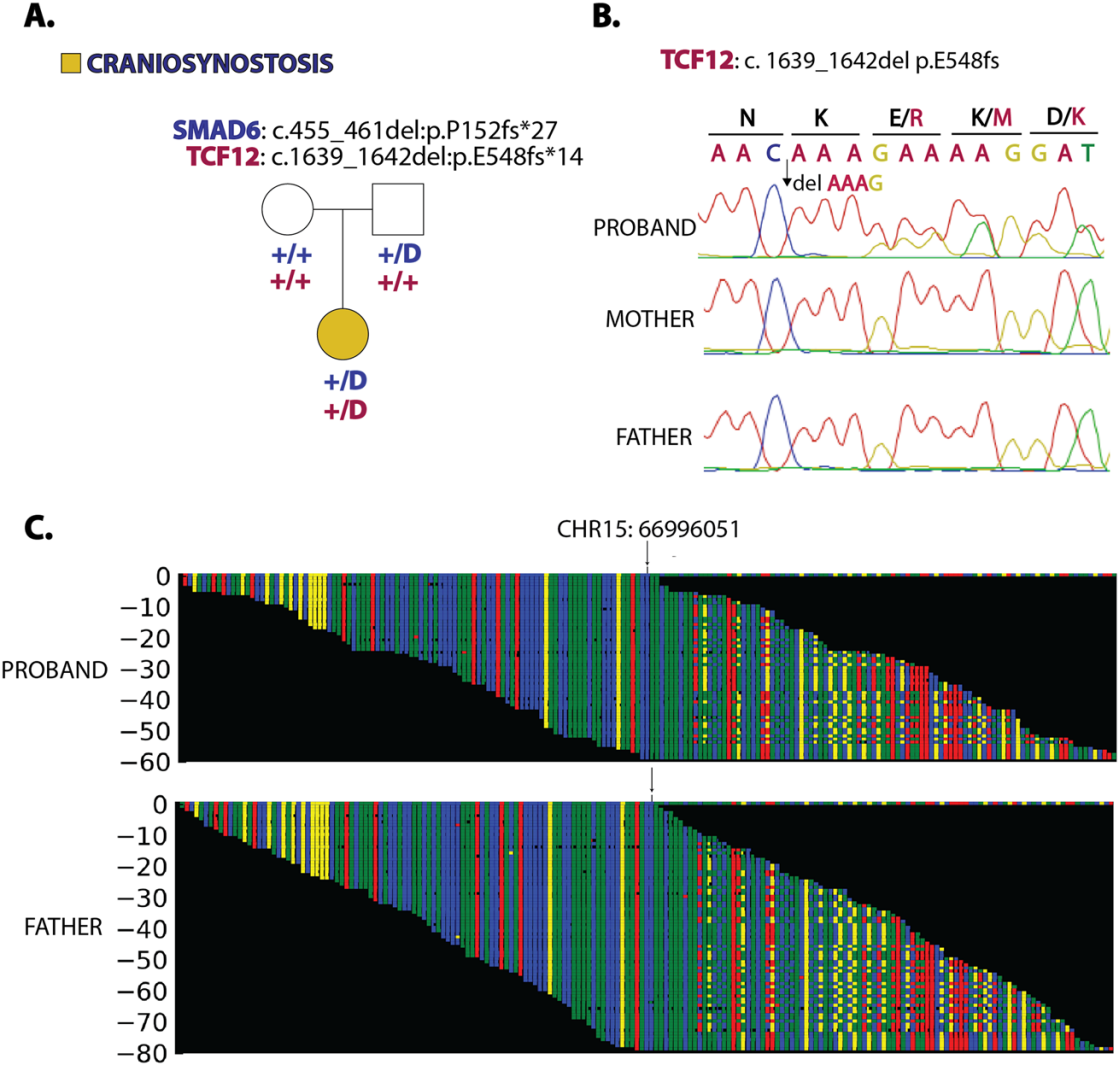
Heterozygous LOF mutations in *SMAD6* and *TCF12* in a proband with complex craniosynostosis. **a** Pedigree and genotypes. Genotypes of each subject are shown: *SMAD6* genotypes are in blue, and *TCF12* genotypes are in red. “+” and “D” denote the wild-type and indicated frameshift alleles, respectively. No member of the trio harbored the *BMP2* risk SNP ‘C’ at rs1884302. The *SMAD6* p.152 fs*27 mutation was transmitted from an unaffected parent, and the *TCF12* p.E548fs*14 mutation arose de novo in the proband. **b** Confirmation of the de novo TCF12 mutation. Sanger sequencing traces of PCR amplicons containing the *TCF12* mutation identified by exome sequencing are shown. The mutation identified in the DNA sequence and its impact on TCF12 protein (in single letter code) are indicated above the trace. The deleted bases are denoted, and they result in an overlap of wild-type and mutant sequences. Both the mother and father’s traces demonstrate the wild-type *TCF12* sequence, whereas the proband has a de novo 4-bp deletion that results in a frameshift. **c** In silico visualization of the *SMAD6* frameshift deletion in the proband. Sequence reads derived from single molecules on the Illumina platform are shown. The reference sequence of a segment of *SMAD6* that includes base 15:66996051 (denoted by arrow) is shown in the top row, and red, blue, green and yellow squares represent the bases A, C, G, and T, respectively. Below, all independent reads that map to this interval are shown. The results show that the proband and father both have a 7-bp deletion that causes a frameshift in the *SMAD6* coding sequence

**Table 1:** Rare loss-of-function variants identified in a child with complex craniosynostosis

Gene name	Chrom	Position	Ref	Alt	Mutation class	Impact	ExAC frequency	pLI
RIIAD1	1	151694016	G	T	stopgain	p. E2X	Novel	NA
ENPP6	4	185033931	AT	—	frameshift deletion	p. M296fs	Novel	0
SMOC1	14	70418934	GCAGGTCCTAC	—	frameshift deletion	p. G60fs	Novel	0.01
TCF12	15	57555366	AAAG	—	frameshift deletion	p. E548fs	Novel	0.97
SMAD6	15	66996051	CGGCGGG	—	frameshift deletion	p. P152fs	Novel	0
ZNF551	19	58199463	G	—	frameshift deletion	p. R579fs	Novel	0

Table containing all rare (ExAC frequency < 2 × 10^−5^) LOF variants identified in a child with complex craniosynostosis. Two novel LOF variants in previously identified craniosynostosis genes (*SMAD6* and *TCF12*) were identified. The novel *TCF12* frameshift mutation was discovered to have arisen de novo in the proband (Fig. 2).

Heterozygous *TCF12* mutations have been previously shown to cause coronal CS with considerable phenotypic overlap with Saethre–Chotzen syndrome^4^, which is caused by LOF mutation in *TWIST1*, which heterodimerizes with *TCF12* to inhibit transcription downstream of BMP signaling. Similar LOF mutations in *TCF12* were subsequently identified in patients with non-syndromic coronal craniosynostosis^6^. De novo or transmitted LOF mutations in *SMAD6* are found in ~6% of non-syndromic midline craniosynostosis cases^5^. LOF mutations in both *TCF12* and *SMAD6* both show striking incomplete penetrance (~40 and 20% penetrance, respectively)^4,7^. In the case of *SMAD6*, epistatic interaction with a common risk variant near BMP2, which by itself has modest effect on risk, increases penetrance to >90%^5,7^. The *BMP2* rs1884302 locus was genotyped in the proband and both parents, and no family members harbored the CS risk allele ‘C’, consistent with the parent harboring the *SMAD6* mutation being free of CS (Fig. 2).

The combination of rare LOF mutations at established Mendelian loci (*SMAD6* and *TCF12*) in the proband was particularly interesting^8^. While *SMAD6* has long been known as an inhibitory-SMAD that negatively regulates BMP signaling, *TCF12* silencing in mesenchymal stem cells was only recently shown to result in increased phosphorylation of receptor-SMADs, implying that loss of TCF12 function also augments BMP signaling via the BMP/SMAD axis^9^. This finding suggests that the combination of *SMAD6* and *TCF12* haploinsufficiency increases BMP signaling to levels substantially greater than those seen with either mutation alone, sufficient to ensure penetrance at both the sagittal and coronal sutures (Fig. 3). Moreover, while neither *SMAD6* nor *TCF12* haploinsufficiency in isolation has been associated with increased rates of reoperation^4,5^, we propose that these mutations together promote sufficiently high osteogenic drive to promote the very unusual rapidity of recurrent synostosis after surgery (Figs. 1, 3).

**Fig. 3:**
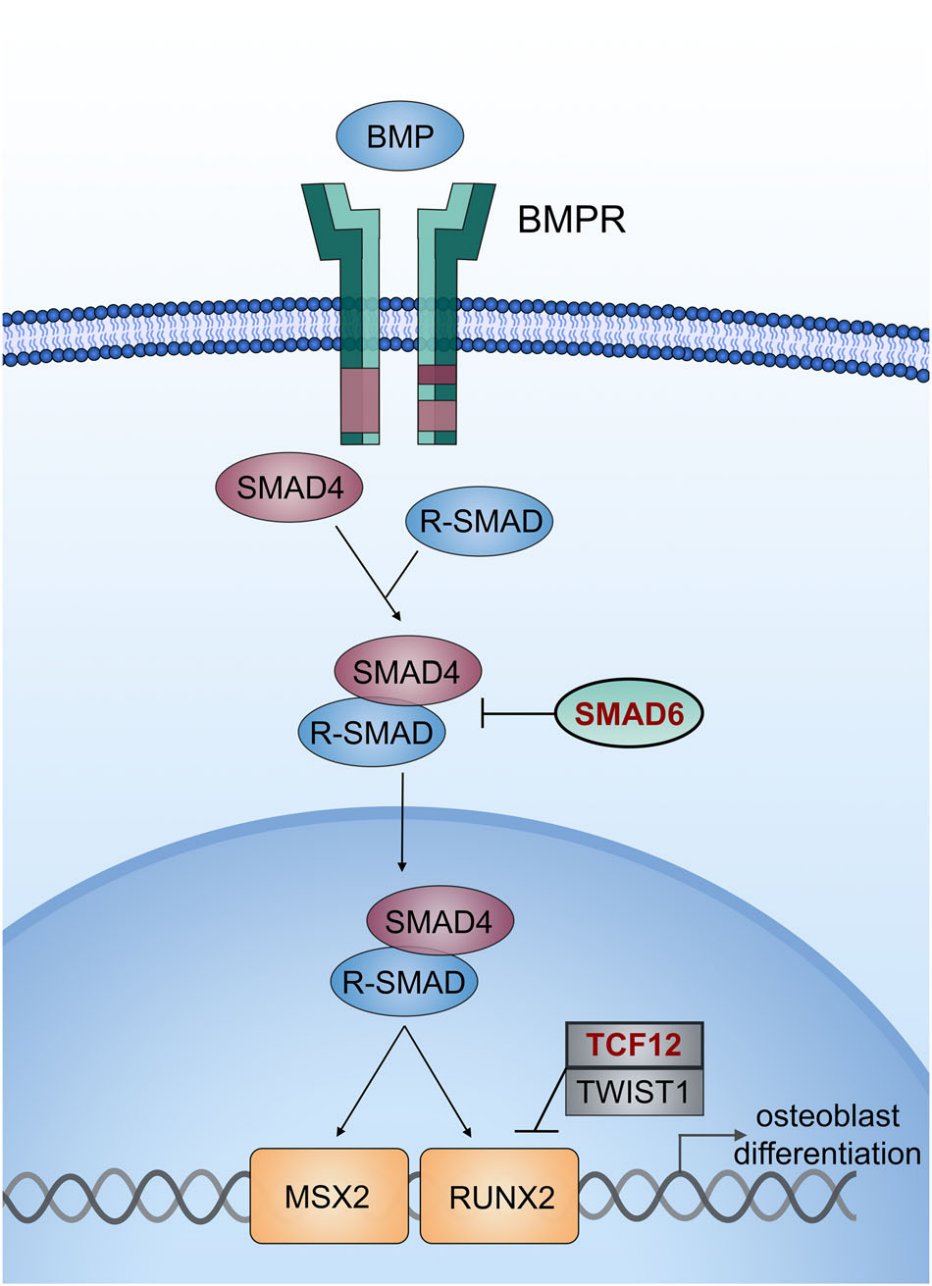
The BMP signaling cascade in osteoblast differentiation. BMP receptors phosphorylate receptor-SMADs upon ligand binding. *SMAD6* is a member of the inhibitory-SMAD family that prevents nuclear translocation of activated SMAD4/receptor-SMAD complexes. *TCF12* and *TWIST1* are basic helix-loop-helix transcription factors that heterodimerize and inhibit transcription downstream of BMP signaling. The co-occurrence of loss-of-function mutations in two master regulators of this signaling pathway that can cause craniosynostosis independently was predicted to result in severely impaired inhibition of BMP/SMAD signaling and excessive osteogenic drive

The combination of a common *BMP2* variant with LOF variants in SMAD6 is sufficient to push BMP/SMAD signaling to levels sufficient to cause suture fusion^5^. The present results suggest that alleles other than the common *BMP2* risk variant can have epistasis with rare *SMAD6* alleles. It seems compelling that the combination of a *SMAD6* LOF mutation with loss of an independent inhibitor of BMP signaling via *TCF12* mutation produces particularly high BMP/SMAD signaling and a strikingly more severe phenotype than *SMAD6* LOF mutation alone (Figs. 1, 3). It will be interesting to see whether other patients with non-syndromic complex CS also have mutations in these two genes.

While several factors, such as patient age at presentation, suture fusion pattern, and patient co-morbidities, play a role in which type of surgery (endoscopic versus open) is offered to craniosynostosis patients, knowing the genetic results for specific patients could prove useful in guiding operative planning for this complex patient population. The goals of cranial vault reconstruction are to obtain an aesthetically pleasing shape of the skull that will allow adequate growth of the brain in ideally one operation. Identification of high risk genotypes prior to surgery—particularly in cases with unusual clinical features—may prove useful in guiding surgical management in the future and may enable more informed discussions with patients’ families.

## HGV Database

The relevant data from this Data Report are hosted at the Human Genome Variation Database at 10.6084/m9.figshare.hgv.2330 10.6084/m9.figshare.hgv.2333.

## Electronic supplementary material

Supplementary Table 1
