# Supplementary material for: Co-occurrence of frameshift mutations in SMAD6 and TCF12 in a child with complex craniosynostosis
Source: Hum Genome Var. 2018 Jun 28;5:14. doi: 10.1038/s41439-018-0014-x (PMC6023907; doi:10.1038/s41439-018-0014-x)
Supplement: Supplementary file 1 — Supplementary Table 1 [file 41439_2018_14_MOESM1_ESM.docx]

**Supplementary Table 1. Rare, damaging missense variants identified in a child with complex craniosynostosis**

| Gene Name | Chrom | Position | Ref | Alt | Mutation Class | Impact | ExAC Frequency | pLI |
| --- | --- | --- | --- | --- | --- | --- | --- | --- |
| CAPN14 | 2 | 31416160 | C | A | nonsynonymous SNV | p. W317C | Novel | NA |
| IQGAP2 | 5 | 75858261 | A | G | nonsynonymous SNV | p. T13A | Novel | 0 |
| DDR1 | 6 | 30857151 | C | T | nonsynonymous SNV | p. R121C | Novel | 0.03 |
| NDUFB9 | 8 | 125555516 | A | G | nonsynonymous SNV | p. Y41C | Novel | 0.04 |
| CHMP5 | 9 | 33270713 | C | T | nonsynonymous SNV | p. T105M | 8.24 x 10^-6^ | 0.26 |
| GAL3ST3 | 11 | 65810785 | G | C | nonsynonymous SNV | p. S163R | Novel | 0 |
| TRAPPC2L | 16 | 88926380 | G | A | nonsynonymous SNV | p. S125N | Novel | 0 |
| CLUH | 17 | 2594967 | G | A | nonsynonymous SNV | p. P1228S | 1.94 x 10^-5^ | 1 |
| MRI1 | 19 | 13879746 | T | G | nonsynonymous SNV | p. V278G | Novel | 0 |
| KMT2B | 19 | 36211829 | T | G | nonsynonymous SNV | p. F427C | Novel | NA |
| TMPRSS6 | 22 | 37465119 | C | T | nonsynonymous SNV | p. E703K | Novel | 0 |

Table containing all rare (ExAC frequency < 2 x 10^-5^) damaging missense variants as called by MetaSVM identified in a child with complex craniosynostosis.
